# Dysregulated hepatic UDP-glucuronosyltransferases and flavonoids glucuronidation in experimental colitis

**DOI:** 10.3389/fphar.2022.1053610

**Published:** 2022-11-04

**Authors:** Wanying Zeng, Xiaojing Liu, Yangyang Wu, Yuting Cai, Zhennan Li, Fei Ye, Yuanhong Sun, Feng Li, Huijie Xing, Shuai Wang

**Affiliations:** ^1^ Institute of Molecular Rhythm and Metabolism, School of Pharmaceutical Sciences, Guangzhou University of Chinese Medicine, Guangzhou, China; ^2^ Department of Rheumatology and Immunology, Union Hospital, Tongji Medical College, Huazhong University of Science and Technology, Wuhan, China; ^3^ Infectious Diseases Institute, Guangzhou Eighth People’s Hospital, Guangzhou, China; ^4^ Institution of Laboratory Animal, Jinan University, Guangzhou, China

**Keywords:** UGTs (UDP-glycosyltransferases), glucuronidation, colitis, DSS, baicalein, puerarin

## Abstract

Glucuronidation catalyzed by UDP-glucuronosyltransferases (UGTs) is one of the most important phase II mechanisms, facilitating drug clearance *via* conjugation of glucuronic acid with polar groups of xenobiotics. Accumulating evidence suggests that IBDs impact drug disposition, but whether and how IBDs regulate UGTs and drug glucuronidation remains undefined. In this study, we aim to investigate the expression of UGTs and drug glucuronidation in experimental colitis. Given that glucuronidation occurs primarily in the liver, we analyzed the mRNA changes in hepatic UGTs with a DSS-induced mouse colitis model. Twelve UGTs were downregulated in the liver of colitis mice including UGT1A1 and UGT1A9 (two representative UGTs). Colitis in mice downregulated UGT1A1 and UGT1A9 in the liver but not in small intestine, colon, and kidney. We also established that the downregulation of UGTs was attributed to the disease itself rather than the DSS compound. Moreover, colitis-reduced UGT1A1 and UGT1A9 lead to dampened baicalein and puerarin glucuronidation. PXR was the only UGT regulator significantly downregulated in colitis mice, suggesting dysregulation of PXR is associated with the downregulation of UGT1A1 and UGT1A9, thereby potentially resulting in dysfunction of baicalein and puerarin glucuronidation. Collectively, we establish that UGTs and glucuronidation are dysregulated in colitis, and this effect may cause variation in drug responsiveness in IBDs.

## Introduction

Inflammatory bowel diseases (IBDs) are characterized by chronic inflammation in the digestive tract. Crohn’s disease and ulcerative colitis are the two main forms of IBDs, affecting different parts of the gastrointestinal tract. Approximately 3.5 million people suffer from IBDs worldwide (Molodecky et al., 2012). Though IBD pathogenesis in the gut tract is well-recognized, the liver function under IBDs remains largely unknown. Recent evidence shows that IBDs are accompanied by liver disorders such as primary sclerosing cholangitis, autoimmune hepatitis, and drug-induced liver damage, revealing that IBDs are closely associated with liver function (Fousekis et al., 2018; Wang et al., 2020). Most drugs pass through the liver, which is the principal site for drug metabolism.

Glucuronidation catalyzed by UDP-glucuronosyltransferases (UGTs) is one of the most important phase II mechanisms in the liver, facilitating drug clearance *via* conjugation of glucuronic acid with polar groups of xenobiotics (Yang N. et al., 2017). Glucuronidation occurs mostly in the liver, as well as extrahepatic tissues such as the small intestine and kidney (Nakamura A. et al., 2008). Dysfunction of glucuronidation can result in various disorders such as neonatal hyperbilirubinemia, Crigler-Najjar, and Gilbert syndrome (Kadakol A. et al., 2000; Wang S. et al., 2019). The mammal UGT superfamily comprises four families including UGT1, UGT2, UGT3, and UGT8 (Meech et al., 2019). The UGT1 family has been extensively characterized, largely because of their important roles in pharmacology and toxicology (Meech et al., 2019).

Accumulating evidence shows dysregulation of drug-metabolizing enzymes in the intestinal mucosa of patients with IBD (Wojtal et al., 2009; Drozdzik et al., 2016; Erdmann et al., 2019). More research has been done to investigate the expression changes in drug-metabolizing enzymes with mouse colitis models^,^ (Kusunoki et al., 2015; Fan et al., 2020; Yang et al., 2021). The regulatory role of experimental colitis on cytochromes P450 (CYPs) enzymes has been extensively characterized. The formation rates of 4′-OH-DCF (known metabolite by CYP2C) and 5-OH-DCF (known metabolite by CYP3A) were much lower in hepatic microsomes of DSS-treated mice than in control mice (Fan et al., 2020). Disturbance of hepatic and intestinal UGTs (e.g., UGT1A1 and 1A6) in rats was observed in trinitrobenzene sulfonic acid-induced colitis (Zhou et al., 2013). However, whether and how glucuronidation activity is affected by colitis remains poorly understood.

In the present study, we aim to investigate the impact of experimental colitis on UGTs and drug glucuronidation. Due to the simplicity and high similarity with IBDs, dextran sulfate sodium (DSS) is frequently used to induce experimental colitis in animals. We firstly investigated gut inflammation on the expression of UGTs with DSS-induced colitis mice. Colitis down-regulates UGT1A1 and UGT1A9 in the liver. We further established that the downregulation of UGTs was attributed to the disease itself rather than the DSS compound by performing experiments with one-day DSS administration and *Il-10* ^*−/−*^ spontaneous colitis model. Moreover, we observed that colitis-reduced UGT1A1 and UGT1A9 led to dampened baicalein and puerarin glucuronidation. Among the UGT1A1 and UGT1A9 regulators, only *Pxr* was significantly downregulated in colitis mice, suggesting dysregulation of PXR in colitis is associated with the downregulation of UGT1A1 and UGT1A9, thereby potentially resulting in dysfunction of baicalein and puerarin glucuronidation.

## Methods

### Materials

DSS (36,000–50,000 MW) was purchased from MP Biomedicals (Irvine, CA, United States). Puerarin and baicalein were purchased from Aladdin Reagents (Shanghai, China). Uridine diphosphoglucuronic acid (UDPGA) and alamethicin were purchased from Sigma-Aldrich (St.Louis, MO, United States). Anti-GAPDH (ab8245), anti-UGT1A1 (ab170858) and anti-UGT1A9 (ab88517) were purchased from Abcam (Cambridge, MA, United States). The secondary antibody was purchased from Huaan Biotechnology (Hangzhou, China). TRIzol reagent, reverse transcriptase mix, and SYBR Green Master Mix were purchased from Vazyme (Nanjing, China). Bicinchoninic acid assay (BCA) protein assay kit was purchased from Thermo Scientific (Rockford, IL, United States).

### Mice

Wild-type mice (C57BL/6) mice were obtained from HFK Biotech (Beijing, China). *Il-10* ^*−/−*^ mice (JAX stock number: 002251) were obtained from Dr. Changhui Liu (Guangzhou University of Chinese Medicine, China) All mice were kept under a 12 h light/12 h dark cycle, and food and water were available *ad libitum*. Male mice or female mice (6 weeks old) were used for DSS-induced colitis. *Il-10* ^*−/−*^ mice were examined after 15 weeks. The procedures for mouse experiments were approved by the Institutional Animal Care and Use Committee of Guangzhou University of Chinese Medicine. To develop a colitis mouse model, mice were exposed to 2.5% DSS in drinking water for 7 days, as described previously (Wang et al., 2018; Wang et al., 2020). All mice were sacrificed on day 7.

### Quantitative polymerase chain reaction

Total RNA was extracted from the colon tissue of mice with TRIzol reagent. cDNA was synthesized from 1 μg of total RNA using the reverse transcriptase mix. For qPCR, the 0.2 ml PCR tube was used to prepare the following reaction system: 2× SYBR qPCR Master Mix (5 μl), forward primer (1 μl), reverse primer (1 μl), and cDNA (3 μl). And all reactions were performed in triplicate. The predenaturation was 95°C for 10 min, followed by 40 cycles of 95°C for 15 s and 60°C for 60 s performed with AriaMx Real-time PCR System (Agilent Technologies, CA, United States). All results were processed by the double-delta method (2^−ΔΔCT^). Primers are provided in Table 1.

**TABLE 1:** Primers used in this study.

Gene name	Forward (5′-3′ sequence)	Reverse (5′-3′ sequence)
GAPDH	CAA​GGA​GTA​AGA​AAC​CCT​GGA	CGA​GTT​GGG​ATA​GGG​CCT​CT
UGT1A9	TTT​CGA​TGT​GTG​CGG​CTT​AAC	GGT​TCC​GAG​TTC​TTT​CCT​TGA​A
UGT1A1	CAC​TGG​CTG​AGT​ATG​CTT​GG	CTT​CTG​GAA​TGG​CAC​AGG​GAA
NRF2	AAT​GTG​CTG​GCT​GTG​CTT​TA	TCT​CCT​CGC​TGG​AAA​AAG​AA
PXR	ACT​GCT​GGG​TTT​GCT​GGG​CGT	GGT​GTG​GTC​CAG​CGC​AGC​GT
CAR	GGGCCTTTGCTACAAGAT	AGG​TTT​TTA​TGG​AAG​TGG​AGG​A
AHR	GGC​TAG​CGT​GCG​GGT​TTC​TC	CTA​GAA​CGG​CAC​TAG​GTA​GGT​CAG
PPAR-α	TAC​ACT​GCT​TCC​TTA​CCG​GC	CTG​AAA​CCC​TCA​CCA​CTG​CT
HNF4α	TGG​ATA​TGG​CCG​ACT​ACA​GC	TCA​GAT​GGG​GAC​GTG​TCA​TT
FXR	TGG​TGA​TGC​AGT​TTC​AGG​GT	AGC​TGG​CTT​CAC​ACT​CAT​CC

### Western blotting

Protein samples were subjected to 10% sodium dodecyl sulfate-polyacrylamide gel electrophoresis, and then transferred to polyvinylidene fluoride membranes (Millipore, Bedford, MA). The PVDF membrane was blocked with 5% nonfat dry milk in Tris-buffered saline with Tween20 (TBST), and further incubated with primary antibodies including anti-GAPDH, anti-UGT1A1, or anti-UGT1A9. Following overnight incubation with the primary antibodies, the membranes were washed with TBST buffer and incubated with the secondary antibodies for 1 h at room temperature and then washed in TBST again. Protein bands were visualized with enhanced chemiluminescence and Omega Lum G imaging system (Aplegen, Pleasanton, CA).

### Glucuronidation assay

Glucuronidation assay is performed as previously described (Xu et al., 2020). In brief, the incubation medium contained microsomes (1 mg/ml), magnesium chloride (0.88 mM), saccharolactone (4.4 mM), alamethicin (22 μg/ml), UDPGA (3.5 mM) and flavonoids (5 μM) in 50 mM potassium phosphate (pH 7.4). After incubation for 30 min, the reaction was terminated by adding ice-cold acetonitrile, followed by vortex and centrifugation (10 min; 13,000 g). All experiments were performed in triplicate. Glucuronides were quantified using a Triple Quadruple LC/MS/MS System (Shimadzu, Kyoto, JP) and a Phenomenex C18 column (2.1 × 100 mm, 2 um). The mobile phases were 0.1% formic acid in acetonitrile (mobile phase B) and 0.1% formic acid in water (mobile phase A). For the determination of baicalein-7-O-glucuronide and puerarin-7-O-glucuronide, the gradient elution program was as follows: 0–1 min, 10% B; 1–2.5 min, 10%–90% B; 2.5–3 min, 90% B; 3–3.5 min, 90%–10% B; and 3.5–5 min, 10% B. The mass spectrometer was operated at the positive ion full scan mode. The precursor-product ion pairs used for multiple reaction monitoring (MRM) of baicalein-7-O-glucuronide and puerarin-7-O-glucuronide were m/z 447/271 and 593/417, respectively.

### Statistical analyses

Data are presented as mean ± SD. Statistical differences between two groups were analyzed by Student’s t-test or non-parametric tests (Mann–Whitney *U* test) as appropriate. The level of significance was set at *p < 0.05.

## Results

### Disruption of hepatic UDP-glucuronosyltransferases in mice with experimental colitis

**FIGURE 1:**
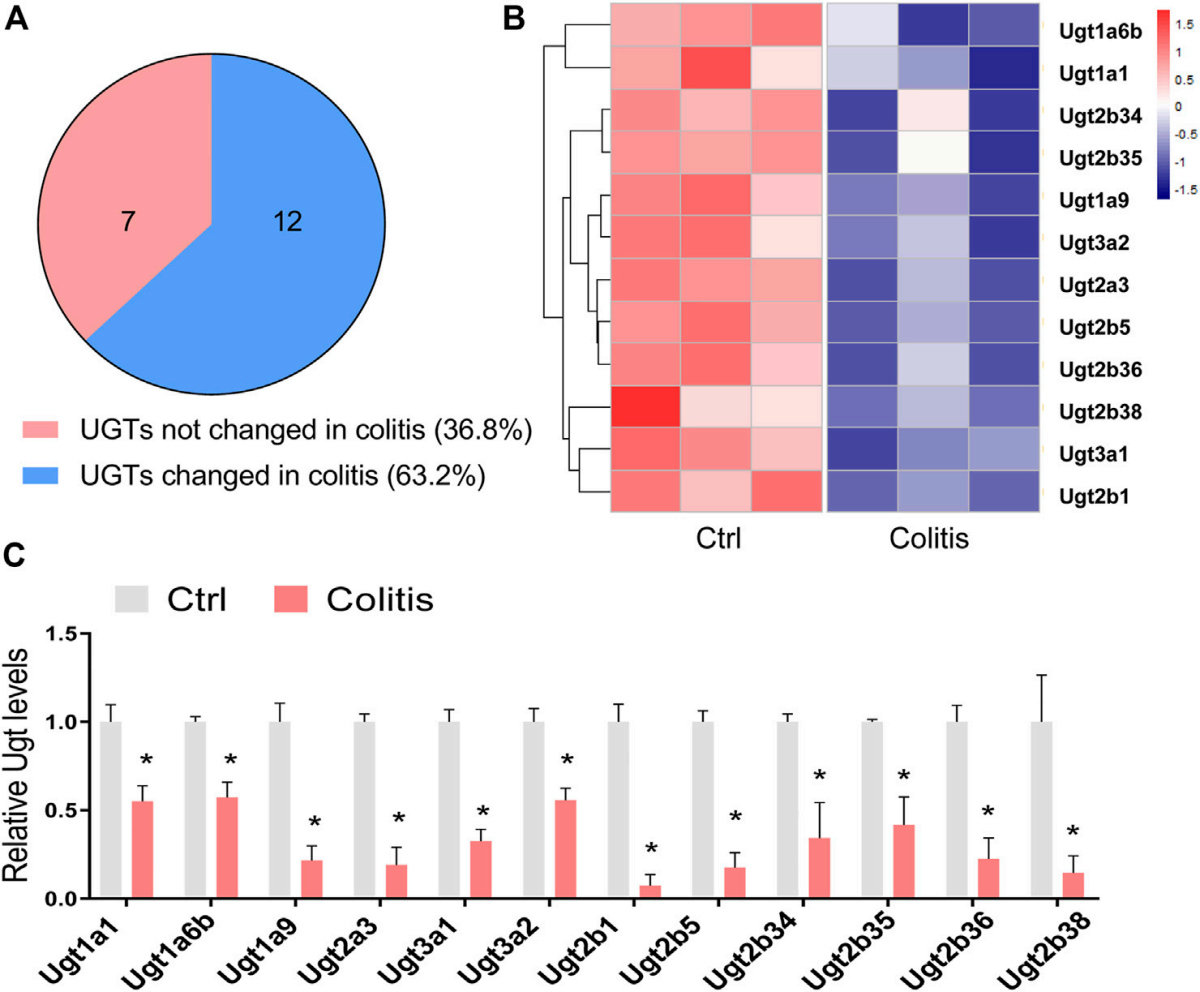
Disruption of hepatic UGTs in mice with experimental colitis. **(A)** Venn diagram shows 12 of 19 UGTs are differentially expressed in colitis. **(B)** Heatmap showing differentially expressed UGTs in the liver of colitis and control (ctrl) mice. **(C)** mRNA expression of differentially expressed UGTs in the liver of colitis and control mice. *p < 0.05.

We firstly examined how colitis affected the expression of hepatic UGTs. We analyzed the mRNA changes in UGTs with a transcriptome dataset (GSE181959) (Zhu et al., 2022). In this study, the 6-week-old mice were treated with 3% DSS for seven consecutive days to induce experimental colitis. We used the R package Limma for differential expression analysis. A total of 19 hepatic UGTs were identified (Figure 1A). Among them, 12 UGTs were downregulated in the liver of colitis mice (Figure 1A). These UGTs include *Ugt1a1*, *Ugt1a6b*, *Ugt1a9*, *Ugt2a3*, *Ugt3a1*, *Ugt3a2*, *Ugt2b1*, *Ugt2b5*, *Ugt2b34*, *Ugt2b35*, *Ugt2b36*, and *Ugt2b38* (Figures 1B,C). This finding indicates that experimental colitis induces dysregulation of UGTs expression in the liver.

### Colitis downregulates UGT1A1 and UGT1A9 in the liver but not in other metabolic tissues

**FIGURE 2:**
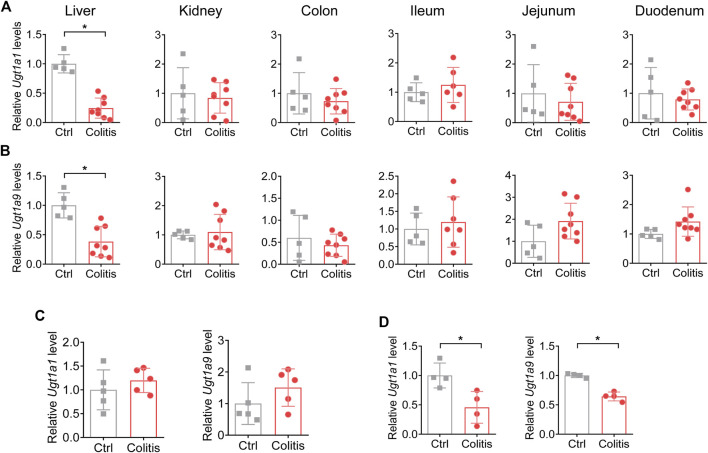
Colitis downregulates UGT1A1 and UGT1A9 in the liver but not in other metabolic tissues. **(A)** mRNA expression of UGT1A1 in the liver, kidney, colon, ileum, jejunum, and duodenum of colitis and control mice. **(B)** mRNA expression of UGT1A9 in the liver, kidney, colon, ileum, jejunum, and duodenum of colitis and control mice. **(C)** mRNA expression of UGT1A1 and UGT1A9 in the liver of mice with 1 day of DSS administration. **(D)** mRNA expression levels of UGT1A1 and UGT1A9 in the liver of *Il-10* ^*−/−*^ and WT mice. *p < 0.05.

Of the UGT family, UGT1A1 and UGT1A9 are two representative isoforms for their abundant expression, significant catalyzing capacities, and clinical relevance (Yuan et al., 2016; Kasteel et al., 2020). Although UGTs mainly catalyze drug glucuronidation in the liver, they are also expressed in extrahepatic tissues such as the kidney, small intestine, and large intestine (Fujiwara et al., 2018). We thus established a DSS-induced colitis model to determine the effects of colitis on UGT1A1 and UGT1A9 in various tissues. Colitis in mice downregulates UGT1A1 and UGT1A9 in the liver but not in other metabolic tissues, indicating colitis-induced dysregulation pattern of UGTs is specifically occurring in the liver (Figures 2A,B).

DSS has been already distributed to the liver 1 day after administration without common features of colitis (Kitajima et al., 1999). To exclude the potential impacts of the DSS compound on UGTs, we further investigated the expression change of UGT1A1 and UGT1A9 1 day after DSS treatment. The results demonstrated that UGT1A1 and UGT1A9 were not changed upon 1 day of DSS administration (Figure 2C). In addition, UGT1A1 and UGT1A9 were downregulated in a genetic colitis model (*Il-10* ^*−/−*^ spontaneously develop colitis), which is in accordance with the results of the DSS-induced colitis model (Figure 2D). We thus proposed that the regulatory effects of colitis on UGTs depend on the disease itself rather than the DSS compound.

### Downregulation of hepatic UGT1A1 and UGT1A9 in colitis leads to dampened glucuronidation

**FIGURE 3:**
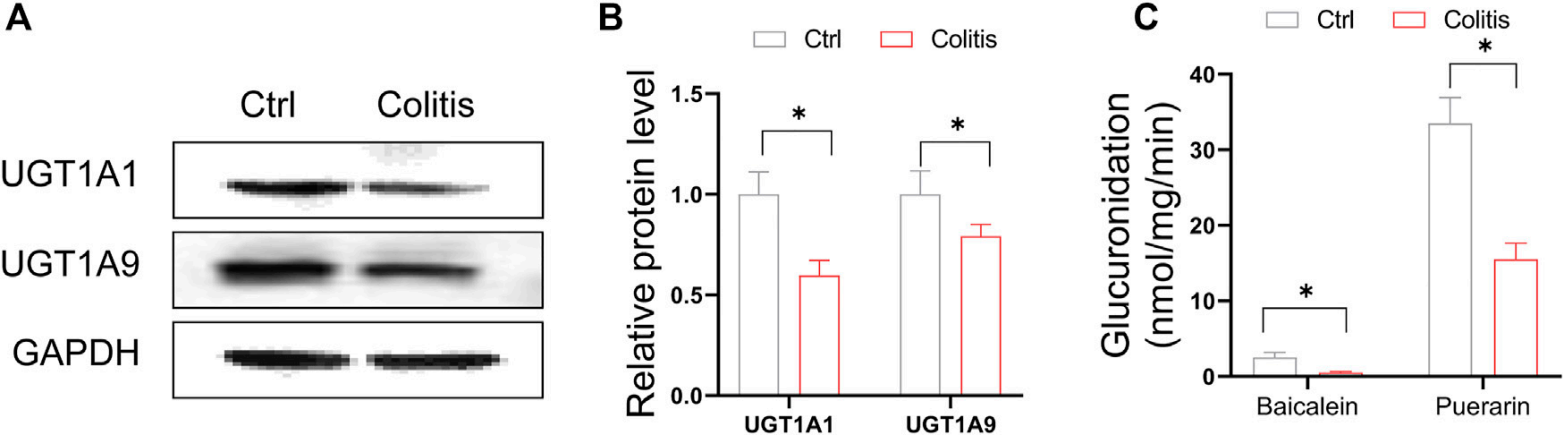
Downregulation of hepatic UGT1A1 and UGT1A9 in colitis leads to dampened glucuronidation. **(A)** Protein expression levels of UGT1A9 and UGT1A1 in the liver of colitis and control mice. **(B)** Quantitative data of Western blotting in **(A)**. **(C)** Liver microsomal metabolism assay showing glucuronidation rate of baicalein and puerarin in colitis and control mice. *p < 0.05.

We confirmed that protein expression of UGT1A1 and UGT1A9 were consistently suppressed in the liver of colitis mice (Figures 3A,B). The expression changes in UGT proteins are significantly associated with the glucuronidation activity of UGT substrates. To explore the impact of DSS-induced colitis on glucuronidation activity, we use baicalein and puerarin as probe compounds to incubate with the liver microsomes of colitis and control mice. Baicalein and puerarin are flavonoids, which are extensively metabolized in hepatocytes by UGTs to form glucuronides. Glucuronidation of baicalein was mainly catalyzed by UGT1A9 (Zhang et al., 2007). And UGT1A1 is the principal enzyme responsible for the formation of puerarin-7-O-glucuronide (a major metabolite of puerarin *in vivo*) (Luo et al., 2012). DSS-induced colitis significantly suppressed the baicalein-7-O-glucuronide and puerarin-7-O-glucuronide formation rate (Figure 3C). Altogether, downregulation of hepatic UGT1A1 and UGT1A9 in colitis leads to dampened flavonoid glucuronidation.

### PXR is associated with the inhibitory effects of colitis on UGT1A1 and UGT1A9

**FIGURE 4:**
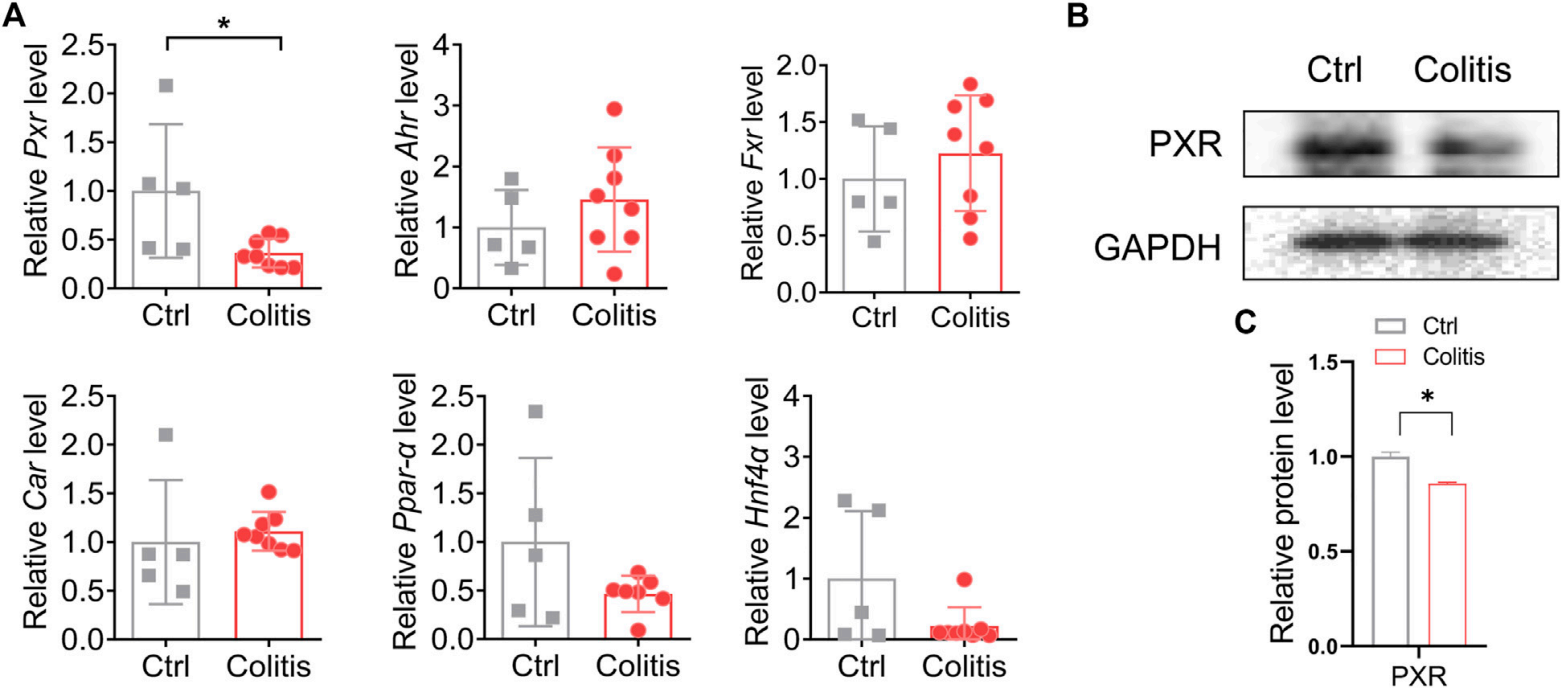
PXR is associated with the inhibitory effects of colitis on UGT1A1 and UGT1A9. **(A)** mRNA expression of PXR, AHR, FXR, CAR, PPARα, and HNF4α in the liver of colitis and control mice. **(B)** Protein expression levels of PXR in the liver of colitis and control mice. **(C)** Quantitative data of Western blotting in **(B)**. *p < 0.05.

Nuclear receptors are a superfamily of ligand-activated transcription factors that regulate the transcription and expression of drug-metabolizing enzymes. UGT1A1 and UGT1A9 have been implicated to be regulated by PXR, AHR, FXR, CAR, PPARα, and HNF4α (Zhou et al., 2005; Mackenzie et al., 2010; Sugatani et al., 2013). We thus investigated how these nuclear receptors mediate colitis-regulated UGT1A1 and UGT1A9 in the liver. Among these nuclear receptors, only *Pxr* was significantly downregulated in DSS-treated mice as compared to control mice (Figure 4A). Moreover, colitis consistently suppressed the protein expression of PXR in the liver (Figures 4B,C). Taken together, these findings indicate that deregulation of PXR in colitis is associated with the downregulation of UGT1A1 and UGT1A9, thereby potentially resulting in dysfunction of flavonoid glucuronidation.

## Discussion

We have observed dysregulation of UGTs and flavonoid glucuronidation in colitis. Downregulation of PXR in colitis is associated with dampened UGT1A1 and UGT1A9, thereby potentially resulting in dysfunction of flavonoid glucuronidation. To exclude the potential impacts of the DSS compound, we investigated the expression change of UGT1A1 and UGT1A9 with two sets of mouse experiments. Firstly, 1 day of DSS administration is insufficient to induce colitis and does not alter the expression of *Ugt1a1* and *Ugt1a9* (Figure 2C). Secondly, *Ugt1a1* and *Ugt1a9* are consistently suppressed in the *Il-10* ^*−/−*^ spontaneously develop colitis mice as compared to the control mice (Figure 2D). Therefore, we propose downregulation of UGTs is attributed to the disease itself rather than the DSS compound. Of note, DSS-induced colitis causes human ulcerative colitis-like pathologies, while *Il-10* ^*−/−*^ mice develop colitis similar to that seen in human Crohn’s disease (Scheinin et al., 2003; Kwon et al., 2021). This finding reveals that the inhibitory effect of colitis on UGTs may present in two forms of IBDs.

It is an interesting finding that all differentially expressed UGTs were suppressed in the liver of colitis mice. It is known that drug glucuronidation is usually catalyzed by one or more UGT enzymes. The overall downregulation of UGTs, therefore, would be detrimental to the metabolism and disposition of a variety of substrates, resulting in the accumulation of the drugs in toxic concentrations. We thus speculate that systematic exposure to these drugs would be enhanced in colitis. Further investigation will be required to clarified the role of colitis on the pharmacokinetics of UGT substrates. In this study, we take UGT1A1 and UGT1A9 as representative examples and investigate the glucuronidation activity using baicalein and puerarin as probe drugs. How colitis impacts glucuronidation activity of other UGT substrates was not addressed in the current study.

Previous studies have established PXR as a positive regulator of UGT1A1 and UGT1A9 (Chen et al., 2003; Duan et al., 2020). In the current study, we observed that the downregulation of PXR in colitis is associated with suppressed UGT1A1 and UGT1A9. We purpose that the production of pro-inflammatory cytokines is elevated in the liver, and subsequently activates Nuclear factor-κB (NF-κB) signaling (Liu et al., 2020; Liu et al., 2021). Further, activated hepatic NF-κB inhibits the nuclear translocation of PXR, resulting in decreased expression and activity of metabolic enzymes (Kusunoki et al., 2014). However, the mechanism by which PXR regulates UGT1A1 and UGT1A9 remains unknown and awaits further exploration.

Noteworthily, UGT1A1 and UGT1A9 display a similar diurnal rhythm in wild-type mice with a peak at midlight phase and a nadir at middark phase (Wang et al., 2019; Xu et al., 2020). Therefore, the inhibitory effects of colitis on UGT1A1 and UGT1A9 could be time-varying. In the current study, the mice were sacrificed at midlight phase. And further experiments were performed at this time point. However, it remains unknown to us whether colitis impacts UGT expression at other time-points, especially in the middark phase. We argue that the time factor cannot be ignored when studying some metabolizing enzymes that oscillate around the clock.

Altogether, experimental colitis induces dysregulation of UGTs and drug glucuronidation in the liver. Dysregulation of PXR in colitis is associated with the downregulation of UGT1A1 and UGT1A9, thereby potentially resulting in dysfunction of baicalein and puerarin glucuronidation. Our findings may provide insights into the mechanism of the variation in drug responsiveness in IBDs.

## Data Availability

The raw data supporting the conclusion of this article will be made available by the authors, without undue reservation.
